# Parental Supervision and Its Impact on Children’s Screen Time, Behavior, and Health Outcomes in Duhok City, Iraq: A Cross-Sectional Study

**DOI:** 10.7759/cureus.79113

**Published:** 2025-02-16

**Authors:** Bashar Mohammed

**Affiliations:** 1 Pediatrics, University of Zakho, College of Medicine, Zakho, IRQ

**Keywords:** behavioral outcomes, children's health, digital screens, parental influence, screen time supervision

## Abstract

Background: The modernization of technology today has raised concerns among parents and health professionals about its probable impact on children's sleep, vision, behavior, and school performance.

Aim: This study explores the effects of digital screen use on children's health and behavior, focusing on screen time duration, parental screen use, device types, and supervision. It examines key risk factors and correlations.

Methods: A descriptive study design was used to explore the impacts of digital screen usage on children's health and behavior. Parents of 580 children aged 1-12 years from Duhok City, Iraq, were included. The data were collected through online questionnaires to parents addressing demographic data, screen time, and physical and behavioral issues. IBM SPSS Statistics for Windows, Version 27.0 (2020; IBM Corp., Armonk, New York, United States) was used to analyze the variables, and a p-value < 0.05 was considered significant.

Results: A total of 580 children were involved in this study. The majority (41.6%) were aged 1-3 years, with 59.7% were male. The phone was the most commonly used (39.0%) device and 38.8% of children owned a digital device; 59.5% of parents reported moderate control over screen time, and most of them (46.4%) had secondary or institute education. Post-device use, 51% of parents reported no change in cognition, but 27.6% reported worse cognition and 41.2% noted increased nervousness. Approximately 45.9% of parents supervised screen time. Supervision did not appear to significantly influence sleep disturbances as compared to unsupervised (39.1% vs.33.1%, p=0.135). However, eye problems showed a significant correlation (66.9% vs. 76.4%, p=0.009). Younger parents (25-35 years) were more likely to monitor screen time (58.6%) compared to older parents (>36 years, 23.3%). Regression analysis confirms parents' own screen time notably predicted children's screen time (B=0.155, p<0.001). These findings highlight the importance of parental behavior in influencing children's screen habits and health outcomes.

Conclusion: This study reveals the significant impact of parental screen time on children's digital habits and health outcomes. The findings suggest that children with supervised screen time have fewer sleep and eye problems. Younger parents are more likely to control their children's screen time, highlighting the role of parental involvement. The results highlight the need for evidence-based guidelines and increased awareness to manage screen time effectively, promoting healthier growth in children.

## Introduction

Today, technology has become a vital part of our lives. With the progression of easily accessible devices like smartphones, iPads, tablets, TVs, and video game consoles, children are increasingly exposed to screens from an early age, raising concerns among parents, educators, and health professionals. The World Health Organization (WHO) has established guidelines recommending limits on the duration of screen usage by children, stressing the potential negative effects on physical, mental, and social development [1].

Evidence suggests that prolonged exposure to digital screens can affect multiple aspects of a child's well-being, such as physical health and social behavior [2,3]. Studies have observed that excessive screen time is associated with various health problems. In particular, prolonged screen exposure is linked to a higher risk of obesity due to sedentary and unhealthy dietary habits. Research has found that children who engage in more than two hours of screen use daily are more likely to exhibit signs of obesity [4-6]. Moreover, screen usage has been associated with irregular sleep patterns, resulting in a decrease in sleep duration and quality, which can lead to behavioral issues and cognitive functioning impairment [4,7,8].

This study aims to examine the complex effects of digital screen use on the health and behavior of children, focusing on factors such as screen time duration, the correlation between parents' screen time usage and their children's screen time, the type of device being used, and parental supervision. By exploring key risk factors and associations, the results will provide evidence-based recommendations for managing screen time in pediatric populations.

## Materials and methods

This was a descriptive cross-sectional study conducted between July and August 2024. This design was chosen to provide a picture of the key variables within the study population at a single point in time. the study was approved by the Institutional Ethics Committee of the University of Zakho College of Medicine (approval number: JULY24/UOZEA38 dated July 24, 2024). Participation in the study was voluntary.

The inclusion criteria comprised parents from Duhok City and its surrounding areas and their children aged 1-12 years who completed the questionnaire. The study excluded individuals whose children did not meet the age criteria. The recruitment of individuals was conducted online through social media platforms and parenting forums to ensure a broad demographic representation. All respondents who fulfilled the inclusion criteria were included in the study without a pre-determined sample size calculation.

Data collection and questionnaire design

Data collection was carried out using a structured, self-administered online questionnaire. The questionnaire was developed in Kurdish to ensure participant understanding and pre-tested with a small sample of 10 participants to ensure clarity and relevance. The English translation is given in the Appendices. Feedback from the pre-test was incorporated to refine the questions and improve the instrument’s reliability. The final questionnaire design included sections on demographic information, daily screen usage duration, and the effects of screen usage on health and behavior. The demographic section analyzed participants' age, gender, educational background, occupation, and geographic location. The daily screen usage section included separate questions for parents and children, requesting the average daily screen time and the various types of devices used such as smartphones, tablets, computers, televisions, and gaming consoles. The effects section focused on health-related issues, including questions on the effect of screen use on physical activity levels, vision-related conditions (e.g., eye strain or dryness), sleep disturbances, cognitive behavior, attention span, academic performance, and emotional regulation.

Data analysis

Data analysis was performed using IBM SPSS Statistics for Windows, Version 27.0 (2020; IBM Corp., Armonk, New York, United States). Descriptive statistics, including frequency, percentage, mean, and standard deviation (SD), were used to summarize demographic characteristics, screen usage patterns, and perceived effects. Inferential statistical methods were employed to test associations between variables. Cross-tabulation was used to explore relationships between categorical variables, while linear regression analysis was applied to evaluate the influence of screen usage on health, vision, sleep, and cognitive behavior outcomes. A p-value of < 0.05 was considered statistically significant.

## Results

Most of the children were male (59.7%), with the majority aged 1-3 years (41.6%) (Table 1). Mobile phones were the most frequently used devices (39%), followed by a combination of mobile phone and TV (29.5%). Only 45.9% of parents supervised screen time, and moderate control is reported in 59.5% of cases. Regarding cognitive and behavioral changes, post device use, 27.6% of children showed worse cognition while 41.2% displayed increased nervousness (Table 2). The mean age of children and parents was 5.21 years (SD = 3.06), and 31.04 years (SD = 5.90), respectively. The average screen time for children was 2.07 hours/day, slightly higher than parental screen time at 1.81 hours/day. 

**Table 1:** Distribution of demographic, behavioral, and health variables related to digital screen use among children (N=580)

Variables		Frequency (Percentage)
Categories of children's age	1-3 years	241 (41.6%)
	4-6 years	180 (31.0%)
	≥ 7 years	159 (27.4%)
Sex	Male	346 (59.7%)
	Female	234 (40.3%)
Parental education level	Primary	77 (13.3%)
	Secondary/Institute	269 (46.4%)
	Graduation	177 (30.5%)
	No school entry	57 (9.8%)
What kind of electronic device is using	Mobile	226 (39.0%)
	Mobile+TV	171 (29.5%)
	TV	135 (23.3%)
	Mobile+Ipad+Playstation	48 (8.3%)
To what extent the use is under your control	Moderate control	345 (59.5%)
	Strict control	139 (24.0%)
	No control	96 (16.6%)
Does your child own a digital device?	Yes	225 (38.8%)
	No	355 (61.2%)
Sleep problems?	Yes	208 (35.9%)
	No	372 (64.1%)
Eye problems	Yes	82 (14.1%)
	No	418 (72.1%)
	Not sure	80 (13.8%)
Cognition changes post-device	Same	296 (51.0%)
	Worse	160 (27.6%)
	Better	124 (21.4%)
Behavioral changes post-device	Overactive	78 (13.4%)
	No changes	121 (20.9%)
	Nervousness	239 (41.2%)
	Decrease appetite	78 (13.4%)
	Better mode	64 (11.0%)
Parent supervising child's screen time	Yes	266 (45.9%)
	No	314 (54.1%)
Using devices for educational purposes?	75%	65 (11.2%)
	50%	175 (30.2%)
	25%	340 (58.6%)

**Table 2:** Descriptive statistics of continuous variables

Variables	Minimum	Maximum	Mean	Std. Deviation
Childs age in years	1	12	5.21	3.06
Parents age in years	18	44	31.04	5.90
Average Time/day given by children to devices in hours	.30	10	2.07	1.62
Average Time/day given by parents to devices in hours	.30	7	1.81	1.28
Number of children using devices	1	5	2.08	1.02

A significant correlation was observed between parental supervision and eye issues (p = 0.009), with 13.7% of unsupervised children experiencing issues compared to 14.7% of supervised children (Tab;e 3). No significant connection was observed between supervision and sleep problems (p = 0.135). 

**Table 3:** Parental supervision of screen time and child health outcomes

Variables		Parental Supervision of Screen Time			P value
		Yes (n=266)	No (n=314)	Total (N=580)	
		Frequency (percentage)	Frequency (percentage)	Frequency (percentage)	
Child experienced sleep problems?	Yes	104 (39.1%)	104 (33.1%)	208 (35.9%)	0.135
	No	162 (60.9%)	210 (66.9%)	372 (64.1%)	
Child experienced eye problems?	Yes	39 (14.7%)	43 (13.7%)	82 (14.1%)	0.009
	No	178 (66.9%)	240 (76.4%)	418 (72.1%)	
	Not sure	49 (18.4%)	31 (9.9%)	80 (13.8%)	

Table 4 shows that parents aged 25-35 years were more likely to supervise screen time compared to older parents aged >36 years (58.6% vs 32.0%, respectively) (p=0.023). Possible reasons could include generational differences in technology familiarity and less awareness of screen time risks. Higher parental education was linked with better screen time monitoring (Graduation level 36.6% vs. No school entry 9.8%) (p=0.007). 

**Table 4:** Association between parental age and education level and monitoring of children's screen time

variables		Monitoring screen time		Total (N=580), n (%)	Chi-square	df	P value	
		Yes (n=266), n (%)	No (n=314), n (%)					
					7.584	2		
Parental age in years	< 25	25 (9.4%)	27 (8.6%)	52 (9.0%)			0.023	
	25-35	156 (58.6%)	217 (69.1%)	373 (64.3%)				
	> 36	85 (32.0%)	70 (22.3%)	155 (26.7%)				
Parental education level	No school entry	30 (11.3%)	27 (8.6%)	57 (9.8%)	12.260	3	0.007	
	Primary	40 (15%)	37 (11.8%)	77 (13.3%)				
	Secondary/Institute	134 (50.4%)	135 (43%)	269 (46.4%)				
	Graduation	62 (23.3%)	115 (36.6%)	177 (30.5%)				

Table 5 reveals that parental screen time is a significant predictor of child screen time (B=0.155, p<0.001), regardless of the level of parent education. 

**Table 5:** Linear regression analysis for predictors of average daily screen time by the child Dependent variable: average daily screen time by the child

Models	B	Std. Error	t	P. value	95.0%CI for B	
					Lower Bound	Upper Bound
(Constant)	2.221	.267	8.314	.000	1.696	2.745
Parental education level	-.017	.051	-.339	.735	-.116	.082
Parental supervision of screen time	.022	.086	.254	.800	-.147	.190
Average daily screen time by the parents	.155	.044	3.511	.000	.068	.242
To what extent does your child use digital devices for educational purposes?	-.050	.062	-.800	.424	-.171	.072

**Figure 1:**
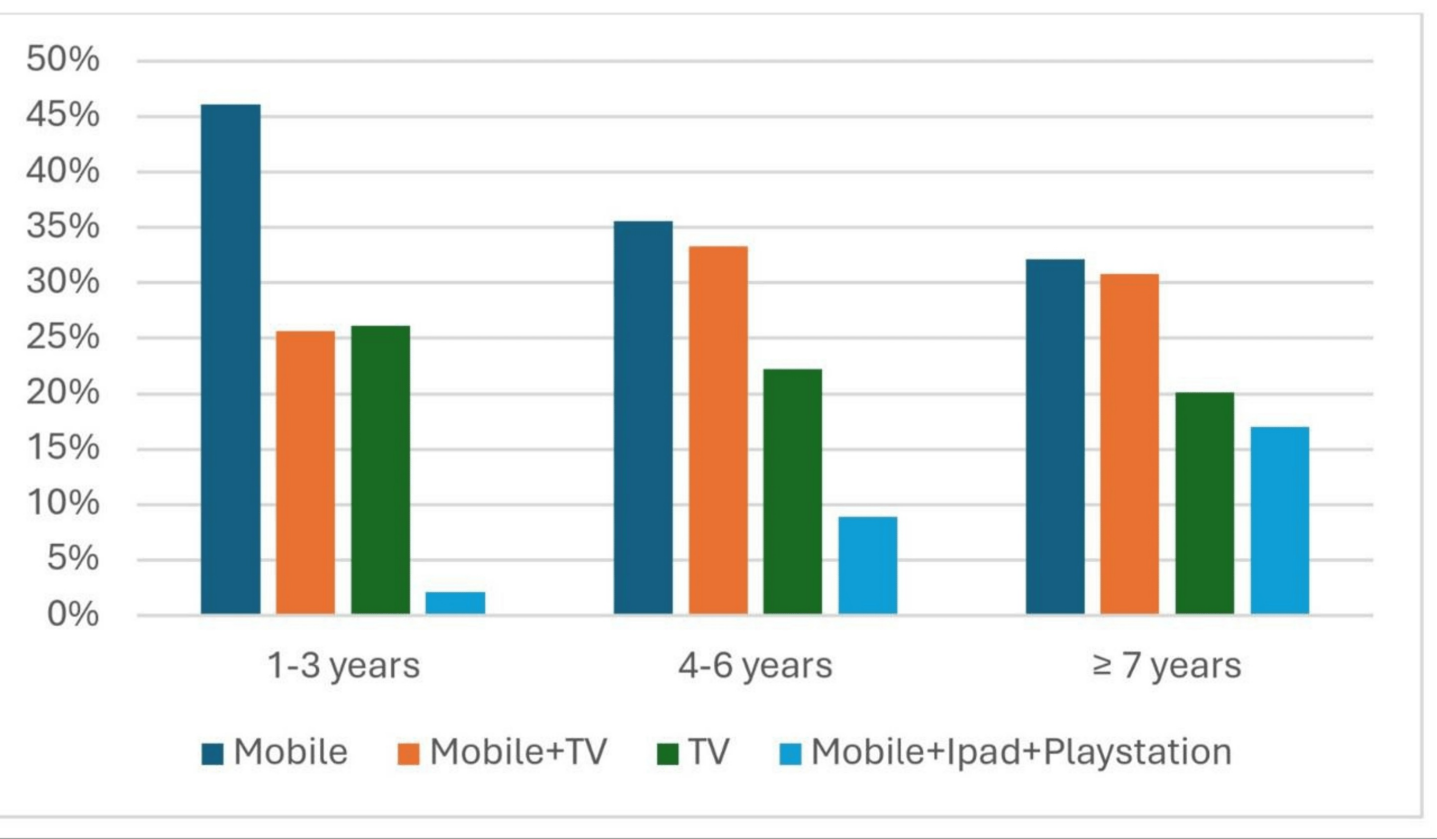
Distribution of device types among children by age group

**Figure 2:**
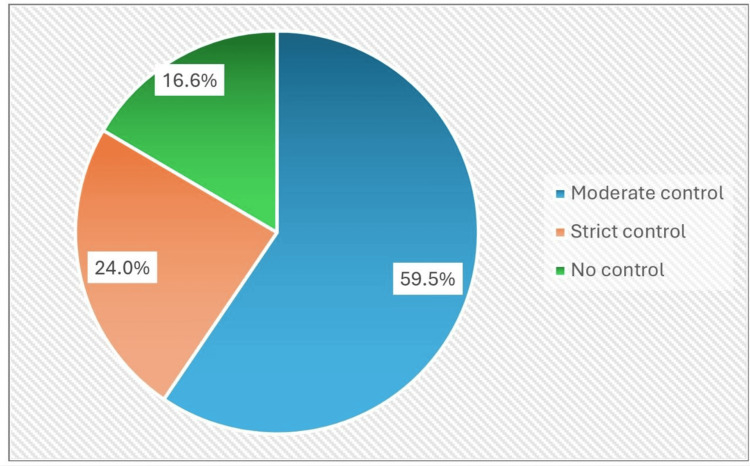
Distribution of parental control levels over children's screen time

Figure 1 illustrates the kinds of devices used by children across different age groups, highlighting the prevalence of mobile phones and TVs combined. Figure 2 shows the levels of parental control over screen time, with moderate control being the most common.

**Figure 3:**
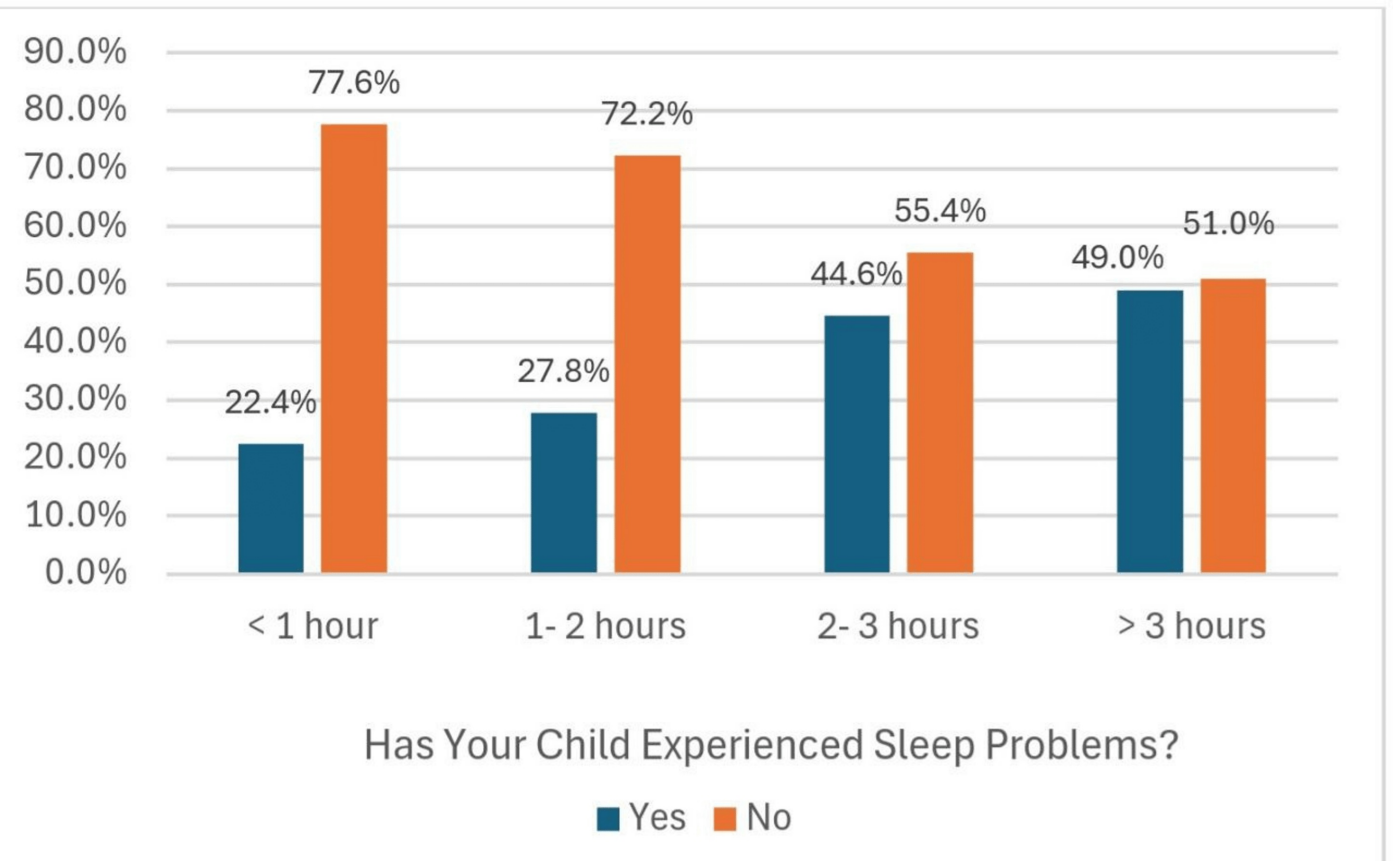
Association of daily screen time with sleep problems

In Figure 3, a clear correlation can be seen between the duration of using screens and sleep problems. The highest percentages pf children with sleep problems (44.6%, 49%) were present in children using screens for two to three hours and more than three hours, respectively. This emphasizes that to minimize sleep problems, screen time should be reduced to less than two hours per day.

## Discussion

This study provides valuable insights into the relationship between parental supervision of children's screen time and its impact on behavior and health outcomes. The results feature several key themes, including the association between screen time and health outcomes, the role of parental education and age in monitoring screen time, and the influence of parental screen time on children's device usage.

Demographic and screen supervision

Our study found that the majority of children were male (59.7%) and aged 1-3 years (41.6%). This aligns with global trends indicating that younger children are progressively exposed to digital devices, with mobile phones being the most frequently used device (39%) [9-11]. In the current study, 45.9% of parents reported controlling their children's screen time, which is consistent with a study in Bangladesh that found 56% of children used screens without supervision, which was associated with higher mental health concerns and behavioral problems [12]. This result is lower than the 65% of United States caregivers who restrict electronics in bedrooms, indicating a gap in supervision practices [13].

Parental influence

In our findings, secondary/institute-educated parents showed a 50.4% monitoring rates that align with other studies [14,15]. Higher maternal education was associated with better screen time management [16,17]. A review conducted by Pons et al. concluded that maternal education directly and indirectly impacts children's recreational screen time (RST) [18]. Although 53% of children had excessive RST (two hours/day), this was lower for ages 2-6 (38%) compared to 6-14 (60%). Also, a study in China by Wang et al. found that higher parental education levels were linked to greater screen accessibility for preschoolers [19]. This contributed to longer screen times, with 43.8% of preschoolers exceeding one hour/day of screen time, particularly in families with higher-educated parents.

Socioemotional effects

In the literature, children with excessive screen time have been found to be related to socioemotional delays, hyperactivity-inattention, and aggressiveness [20-22]. These results are consistent with the present study, which found that 41.2% and 13.4% of children exhibited nervousness and overactivity, respectively, after using digital screens.

Sleep and vision problems

In the current study, 35.9% of the children had sleep problems, with the majority (44.6%, 49.0%) using screen time for two to three hours and more than three hours, respectively. This aligns with other studies that that one extra hour of screen time is associated with a 10-minute decrease in sleep duration [23,24]. Low screen time (<1 hour/day) shows better sleep efficiency (90%) compared to high screen time (>3 hours/day), which is connected with significantly lower sleep efficiency (75%) and more frequent nocturnal awakenings [25-27]. Eye problems were reported in 14.1% of participants, which is consistent with a study by Wang et al., who observed that 15-20% of children who used digital devices for longer than two hours/day experience symptoms of digital eye strain [19].

Interestingly, in a 2025 study by Zhang et al., it was observed that children under supervision exhibited a slightly higher prevalence of eye problems (14.7% vs 13.7%), as parents often encourage the use of educational apps or online learning platforms [28]. In the current study, there is a direct relationship between parents' and kids’ screen time; for each additional unit of parents' screen time, children's screen time increases by 0.155 units. This agrees with the studies by Zhou et al. [29] and Zong et al. [30].

Limitations and future recommendations

There are some limitations of this study. As this was a questionnaire-based study, parent-reported data on screen time and outcomes may be influenced by recall inaccuracies. The cross-sectional design restricts causal inferences and longitudinal research is necessary to explore causal connections and long-term consequences of screen time on health and behavior. Diverse samples (age, gender, culture) should be included for a broader application. Future studies should explore the balance between the benefits and drawbacks of supervised screen use, especially its impact on education and health, to develop evidence-based recommendations for parents

## Conclusions

This study focused on the critical role of parental supervision and education in managing children's screen time and its associated health issues. The duration of children's screen time was directly related to parental screen time. While screen time was dominant, especially among younger children, less than half of the parents supervised it effectively. Higher parental education correlated with better screen time regulation, underscoring the need for increased awareness and education on its risks. Excessive screen time was associated with cognitive decline, behavioral changes, and sleep disturbances in children. The study also revealed that both supervised and unsupervised screen use can contribute to eye problems, indicating that the nature of screen exposure, including educational content, could be a factor. Older parents, especially those above 36 years of age, demonstrated a higher tendency to monitor their children’s screen time compared to younger parents, suggesting that parental age may influence monitoring behaviors.

## Figures and Tables

**Figure 4:**
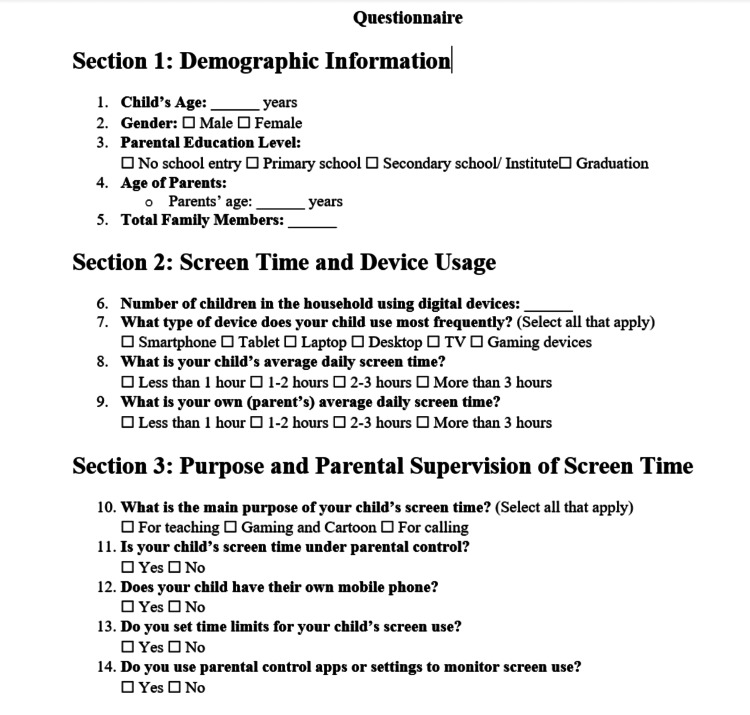
Page 1

**Figure 5:**
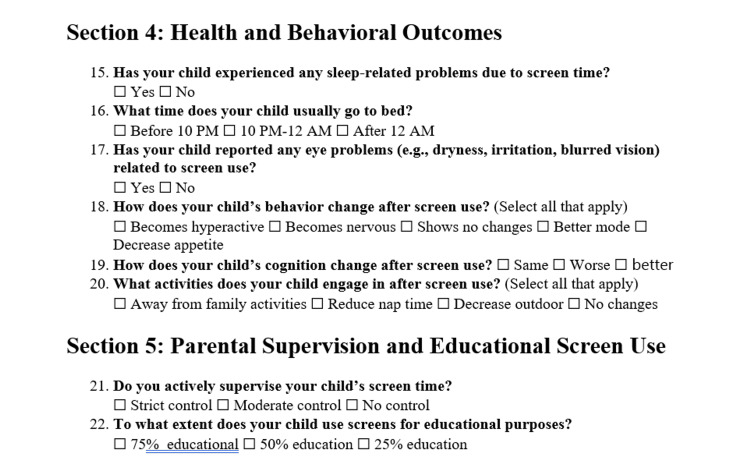
Page 2

## Appendices

Questionnaire (English translation)
